# Hemodynamic effect of coil packing density after tubridge flow diverter for anterior circulation small and medium-sized aneurysms

**DOI:** 10.1038/s41598-026-49706-6

**Published:** 2026-04-24

**Authors:** Yeqing Jiang, Ligang Xu, Qimin Zhang, Guanghu Xu, Long Yu, Xinzhuo Li, Xiaolong Zhang, Jun Wan, Shengzhang Wang

**Affiliations:** 1 Department of Radiology, Huashan Hospital Affiliated to Fudan University, Shanghai, China; 2 Department of Interventional Radiology, Jing’an District Central Hospital, Fudan University, Shanghai, China; 3 Institute of Biomechanics, Department of Aeronautics and Astronautics, Fudan University, Shanghai, China; 4 Academy for Engineering and Technology, Fudan University, Shanghai, China

**Keywords:** Tubridge Flow diverter, Anterior circulation, Intracranial aneurysm, Packing density, Hemodynamics, Diseases, Engineering, Medical research, Neurology

## Abstract

Investigating the impact of coil embolization rate on hemodynamic parameters within the aneurysm sac after treating anterior circulation small and medium-sized aneurysms with the Tubridge (TB) flow diverter. Twenty-six patients with 29 intracranial aneurysms were collected between September 2020 and December 2022. The finite element method simulated preoperative conditions, after TB deployment alone, and with TB plus different coil embolization rates. Different hemodynamic parameters were analyzed under these treatment scenarios. Variance analysis and multiple comparisons were conducted to find an appropriate embolization rate. Twenty-nine aneurysms from 26 patients showed, with increasing TB deployment and coil embolization rates, a continuous improvement in the aneurysm sac’s Qinflow, Va, WSS, and normalized and ratio residual blood volume (nRFV and rRFV) under different thresholds (0.1 m/s, 0.15 m/s). Quantitative analysis indicated the impact on hemodynamics was not significant (*P* > 0.05) when the coil embolization rate reached 5% and beyond for Qinflow, Va, WSS, and RFV under different thresholds (0.1 m/s, 0.15 m/s). Adjunctive embolization can improve the hemodynamic environment within the small and medium-sized aneurysm sac when using the Tubridge flow diverter. However, once the embolization rate within the sac reaches 5%, the hemodynamic environment tends to stabilize, making dense embolization unnecessary.

## Introduction

The Tubridge (TB) is a specialized woven-stent flow diverter (FD) designed to redirect blood flow from intracranial aneurysm sac back into the parent artery. Its deployment represents a novel treatment strategy for intracranial aneurysms, focusing primarily on arterial reconstruction through hemodynamic alteration. This approach signifies a shift in paradigm from intrasaccular embolization to flow reconstruction in aneurysm treatment, achieving occlusion rates exceeding 90% during follow-up^1^. TB demonstrates significant advantages over traditional coil embolization, especially in treating large and giant aneurysms^2,3^. Emerging evidence also supports its efficacy in small and medium-sized aneurysms^4^.

However, considerable variability exists in the timing and degree of aneurysm occlusion among individuals. While the use of Pipeline Embolization Devices (PEDs) does not necessitate adjunctive coil embolization, an increasing number of neurointerventional specialists are combining TB deployment with coils. This combined approach has demonstrated superior efficacy in achieving aneurysm occlusion compared to TB alone^5,6^. Dense packing is standard practice with conventional coil embolization or stent-assisted coiling; however, no established guidelines exist for packing density (PD) when using TB, where loose packing is typically employed. From a hemodynamic perspective, additional coils may not be necessary if the flow within an unruptured aneurysm sac remains stagnant. Furthermore, more intrasaccular manipulation increases the risk of intraoperative rupture, as well as treatment costs. A previous study investigating optimal packing density in large aneurysms treated with pipeline embolization devices assisted coiling simulation reported a value of 7.5% via hydrodynamic variation^5^. However, the optimal packing density for coil embolization following TB deployment in small and medium-sized aneurysms remains to be determined.

Therefore, we investigated the hemodynamic changes of adjunctive coil embolization followingTB deployment to identify the optimal packing density for achieving effective occlusion with loose packing. We hypothesized that beyond a specific PD threshold, additional coils would not confer further hemodynamic benefits. This study aimed to explore optimal coil embolization strategies following the use of FDs.

To this end, we utilized patient-specific aneurysm models derived from 26 consecutive patients (29 aneurysms) treated at our center, capturing pre- and post-intervention states. Finite Element Analysis (FEA) was then employed to construct high-fidelity models, enabling precise post-treatment Computational Fluid Dynamics (CFD) analyses^7^. These models simulated the virtual treatment process involving Tubridge (TB) deployment followed by coil embolization at varying packing densities. Assuming TB implantation, we further analyzed the hemodynamic effects associated with different coil packing densities.

## Results

**Fig. 1:**
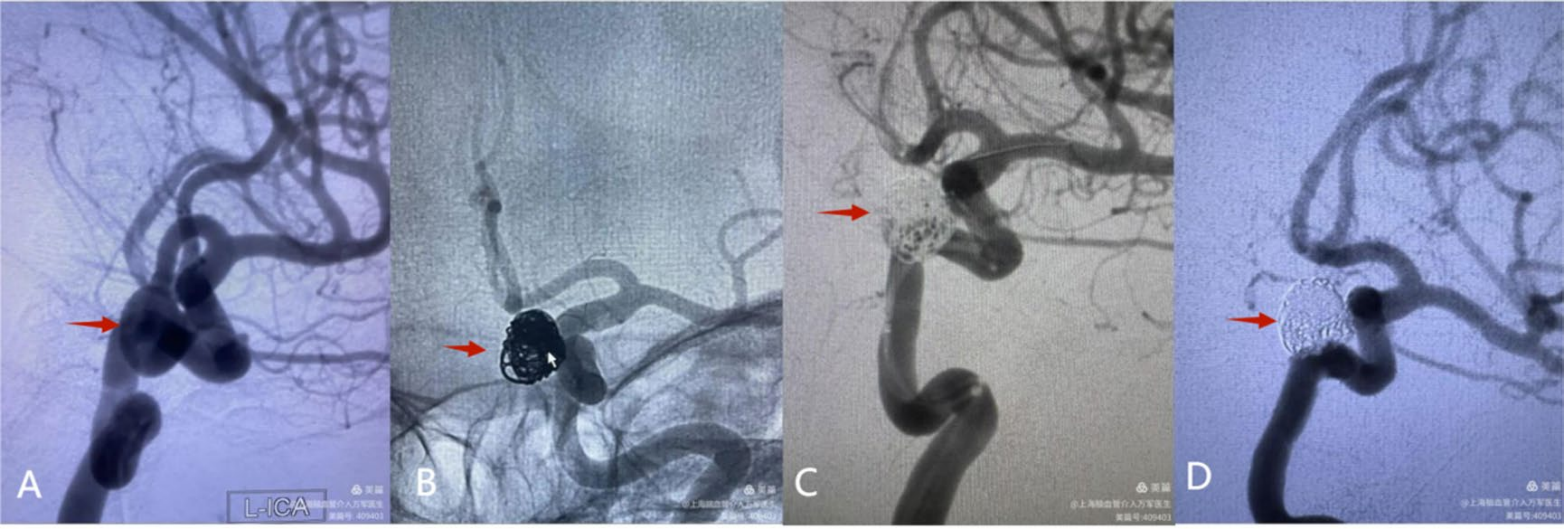
An elderly female patient with a left carotid-ophthalmic aneurysm was treated with a 4–20 mm Tubridge (red arrow means anterior wall aneurysm). (**A**): Preoperative DSA image; (**B**), (**C**): Postoperative DSA images; (**D**): DSA angiogram at 21 month follow-up showed complete occlusion of aneurysm.

**Fig. 2:**
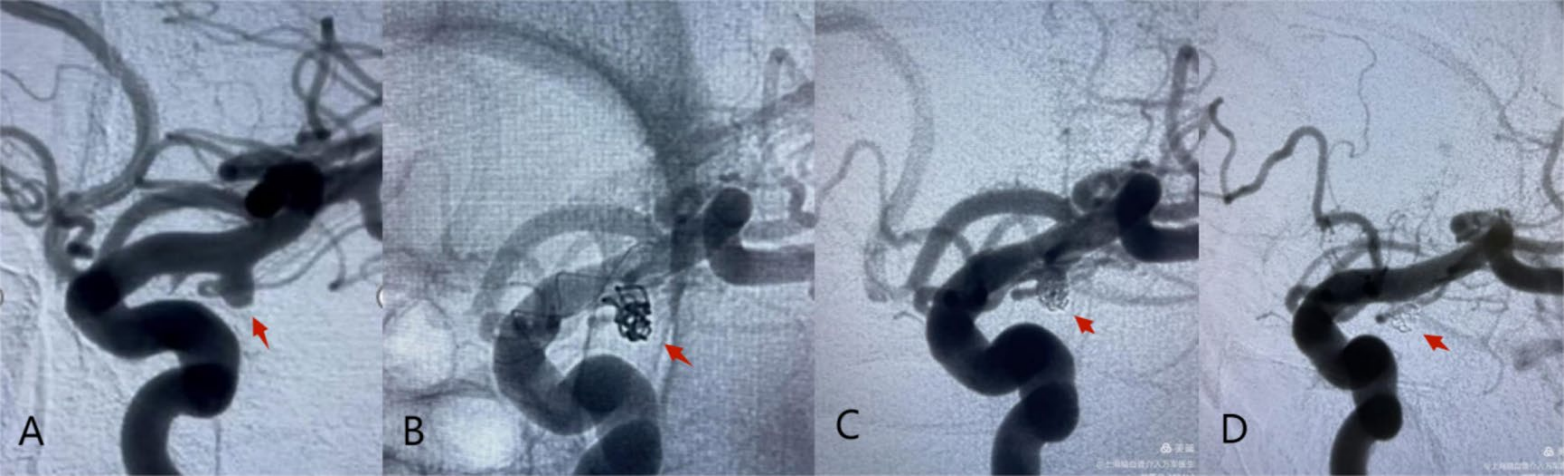
An 58-year-old male patient with a small left internal carotid artery posterior communicating artery aneurysm was treated with a 4.0–15 mm Tubridge assited with loosen coils(red arrow means aneurysm). (**A**): Preoperative DSA image; (**B**), (**C**): Postoperative DSA images; (**D**): DSA angiogram at 27 month follow-up showed complete occlusion of aneurysms.

A total of 29 aneurysms derived from 26 patients were enrolled in this study, all of which were unruptured aneurysms. Among them, 28 were saccular aneurysms, and 1 was fusiform. The cohort consisted of 10 male and 16 female patients, with an average age of 58.38 ± 11.34 years. Smoking was reported in 6/26 cases (23.1%), alcohol consumption in 3/26 cases (11.5%), hypertension in 11/26 cases (42.3%), and diabetes in 3/26 cases (11.5%). Aneurysm locations included: 16 at the internal carotid-ophthalmic segment (Fig. 1), 4 at the ICA cavernous segment, 4 at the ICA posterior communicating segment (Fig. 2), 2 at the ICA anterior choroidal artery involved, and 3 at the middle cerebral artery. Branch involvement at the aneurysm neck was observed in 7 cases: 2 at the posterior communicating artery, 2 at the ophthalmic artery, 1 at the M1 branch, and 2 at the anterior choroidal artery. The aneurysm neck measured 4.62 ± 1.92 mm (range: 2–9 mm), with a mean height of 4.88 ± 3.47 mm and a mean width of 4.94 ± 2.97 mm.

### Qualitative analysis

**Fig. 3:**
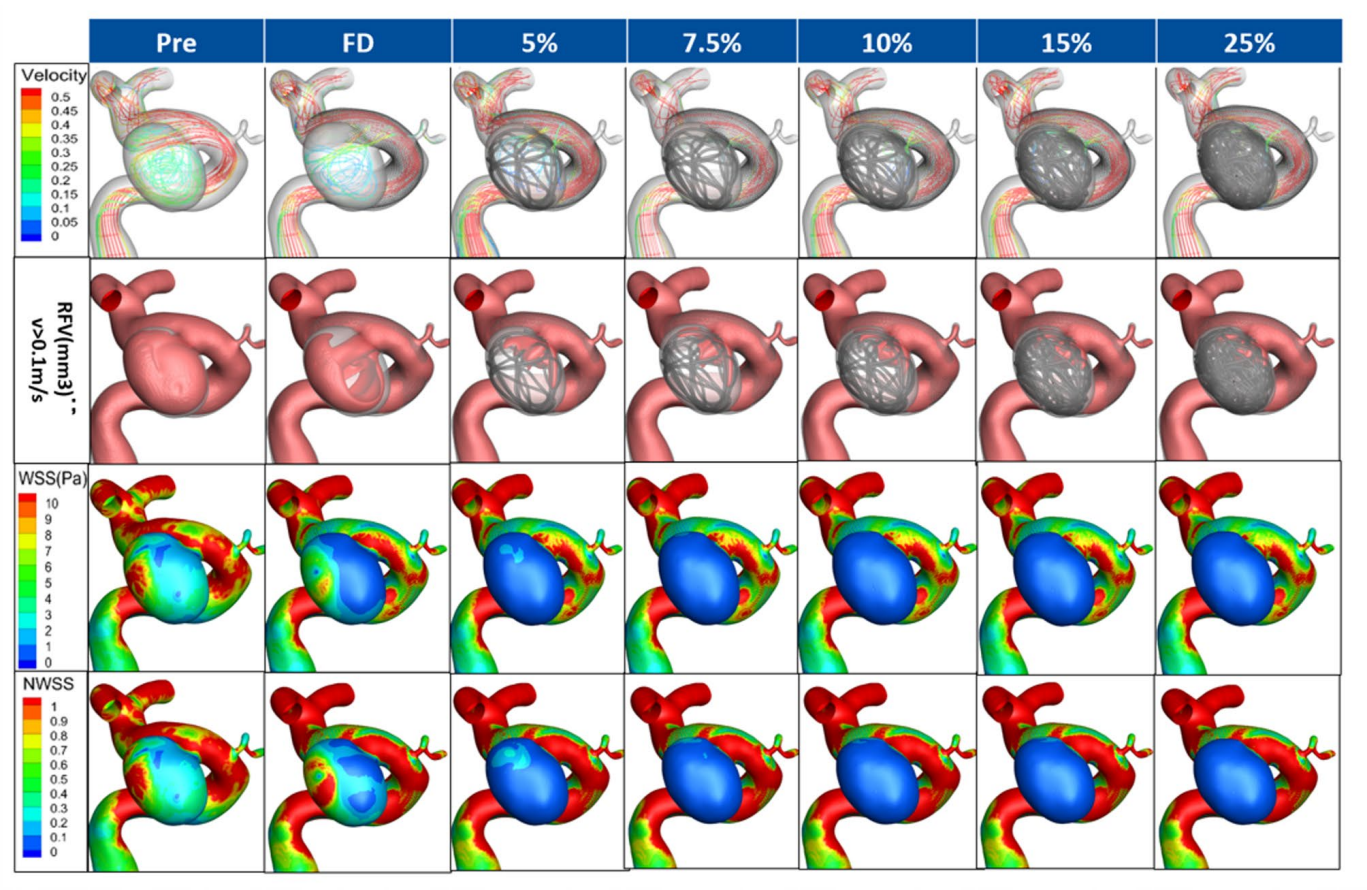
Illustrations of blood flow velocity streamlines(m/s), residual blood flow volume(v>0.1 m/s), wall shear stress, and normalized wall shear stress in a case of carotid-ophthalmic aneurysm before operation, after simple TB, and under different embolization rates (pre-operation, after TB, coiled 5%, 7.5%, 10%, 15%, 25%). Hemodynamic parameters within the aneurysm sac tend to stabilize when the embolization rate exceeds 5%.

**Fig. 4:**
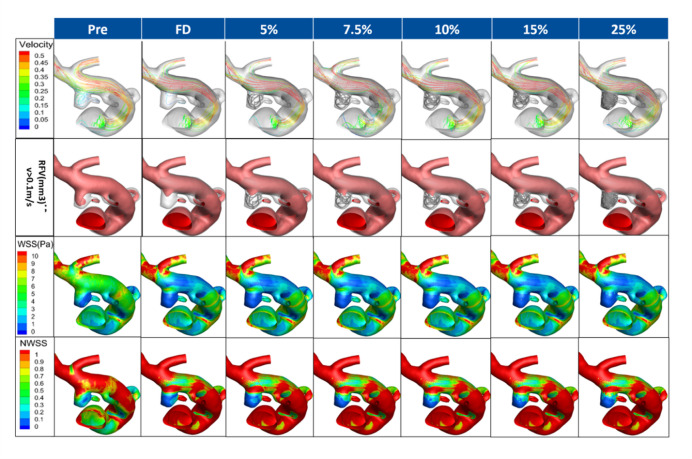
Illustrations of blood flow velocity streamlines(m/s), residual blood flow volume(v>0.1 m/s), wall shear stress, and normalized wall shear stress in a case of posterior communicating artery aneurysm before surgery, after simple TB, and under different embolization rates(pre-operation, after TB, coiled 5%, 7.5%, 10%, 15%, 25%). Hemodynamic parameters within the aneurysm sac tend to stabilize when the embolization rate exceeds 5%.

Qualitative analysis of 29 aneurysms in 26 patients indicates that as the TB stent implantation and coil embolization rate increase, there is a progressive improvement in the inflow volume into the aneurysm sac, the average flow velocity within the aneurysm, the wall shear stress (WSS) on the aneurysm wall, the average wall shear stress, and the residual blood flow volume under different thresholds (V = 0.1 m/s,0.15 m/s,). Typical case 1 (Fig. 3) showed that a internal carotid-ophthalmic artery aneurysm was treated via FD and different coil packing density, the flow jet was diminished, especially after the coil insertion, residual flow volume and wall shear stress gradually decreased. And case 2 (Fig. 4) demonstrated a posterior communicating artery (PcomA) aneurysm. The aneurysm neck involved the PcomA. The hemodynamic parameters also showed progressive improvement.

### Quantitative analysis

Twenty-six consecutive patients with 29 intracranial aneurysms underwent hemodynamic simulation using a combination of flow diverter (FD) stent and flow-reducing coils. The computational models included preoperative baseline conditions, FD stent placement alone, and FD stent combined with flow-reducing coils at five different embolization rates (5%, 7.5%, 10%, 15%, and 25%). Key hemodynamic parameters were systematically evaluated including the Q_inflow_, Va, the AnWSS, NWSS and nRFV and rRFV calculated with average flow velocity of greater than 0.1 m/s, 0.15 m/s, respectively. Statistical analysis revealed significant packing density-dependent improvements in all evaluated parameters following therapeutic intervention (*p* < 0.0001 for all comparisons, one-way ANOVA). Post-hoc analysis demonstrated marked hemodynamic reduction after FD stent + plus 5% packing density placement compared to baseline (*p* < 0.05 for all parameters, student-Nweman-keuls, SNK). No significant differences between incremental embolization rates of 5%, 7.5%,10%, 15% and 25% with progressive parameter reduction followed by stabilization after 5% embolization (Table 1).

**Table 1:** Calculated values and relative changes of hemodynamic parameters under different embolization rates, results of variance analysis, and multiple comparisons.

CFD parameters	Values under the different packing density(mean)							Relative changes caused by different embolization rates (compared to preoperative values)						ANOVA	Multiple Comparisons—*P*-Value				
	Pre	FD	5%	7.5%	10%	15%	25%	FD	5%	7.5%	10%	15%	25%	*P*-value	FD vs. 5%	5% vs. 7.5%	7.5% vs. 10%	10% vs. 15%	15% vs. 25%
Q_inflow_(ml/s)	1.598	1.078	0.869	0.805	0.782	0.699	0.583	31.82%	49.01%	53.23%	55.51%	62.97%	71.08%	<0.0001	0.020	1.000	1.000	1.000	1.000
Va(m/s)	0.308	0.098	0.057	0.049	0.045	0.036	0.029	48.79%	70.42%	74.58%	76.02%	81.07%	85.10%	<0.0001	<0.0001	0.974	1.000	0.674	0.894
AnWSS(Pa)	3.577	1.689	1.077	0.956	0.903	0.830	0.755	52.48%	69.56%	73.21%	74.60%	76.20%	78.64%	<0.0001	0.001	0.996	1.000	1.000	0.990
NWSS	0.676	0.497	0.347	0.315	0.299	0.288	0.261	24.93%	50.29%	54.74%	57.17%	59.04%	62.66%	<0.0001	0.001	1.000	1.000	1.000	1.000
nRFV(mm^3^): v>0.1 m/s	131.23	64.382	24.500	18.411	16.951	10.958	7.987	45.65%	79.15%	83.77%	86.36%	91.18%	93.61%	<0.0001	0.002	1.000	1.000	0.959	1.000
nRFV(mm^3^): v>0.15 m/s	95.961	34.833	11.794	9.574	8.732	5.983	4.726	67.19%	88.14%	90.23%	91.77%	94.41%	95.61%	<0.0001	0.031	1.000	1.000	0.998	1.000
rRFV: v>0.1 m/s	62.42%	33.82%	13.03%	10.52%	8.99%	6.16%	4.47%	46.63%	79.64%	83.56%	85.85%	90.19%	92.61%	<0.0001	0.001	1.000	1.000	0.987	1.000
rRFV: v>0.15 m/s	49.16%	17.33%	6.01%	5.25%	4.35%	2.93%	2.29%	67.72%	88.45%	89.98%	91.32%	93.77%	94.85%	<0.0001	0.025	1.000	1.000	1.000	1.000

Qinflow: the inflow rate at the neck; Va: the averaged velocity of the aneurysm sac; AnWSS: aneurysmal WSS; NWSS: the normalized WSS; nRFV: the normalized residual flow volume; rRFV: the ratio of the RFV to the aneurysm sac volume.

### Clinical results

All packing density were large than 5% for 16 (16/29, 55.2%) aneurysms with Tubridge stent combined with adjunctive coiling. Four patients with 6 aneurysms refused follow-up, of which 2 aneuerysms with Tubridge stent combined with adjunctive coiling. Twenty-three aneurysms(23/29, 79.3%) were followed up (20.13 ± 9.69 months, 3–41 months), among which 14 aneurysms were in the Tubridge combined with adjunctive coiling group (packing density > 5%, 24.3 ± 15.5%, range 11.5–68.6%), of which only 1 aneurysm (7.1%) remained unhealed. Among the 9 aneurysms in the only Tubridge stent group, 6 aneurysms (66.7%) remained unhealed. there was a statistical difference between the two groups (*P* = 0.005).

## Discussion

This study provides valuable insights into the hemodynamic effects of varying adjunctive coil packing densities (PD) following Tubridge flow diverter (TFD) deployment in the treatment of small and medium-sized anterior circulation aneurysms. By combining finite element analysis (FEA) and computational fluid dynamics (CFD), the research highlights the critical relationship between coil density and the stabilization of hemodynamic parameters within the aneurysm sac. As the packing density (PD) increases, the blood flow velocity within the aneurysm gradually decreases, with diminishing marginal returns for incremental coil insertion beyond a threshold. However, excessive packing in unruptured aneurysms treated with flow diverters may yield negligible hemodynamic benefit while potentially increasing intraoperative rupture risk. For aneurysms with higher PD, this may represent an overzealous approach, as flow diverters possess excellent flow diversion capabilities. Therefore, this study aims to determine the critical threshold for effective hemodynamic benefits during the aneurysm packing process through high-fidelity stent virtual release and porous medium equivalent coil virtual release algorithms, thereby avoiding unnecessary packing after achieving optimal PD.

Computational Fluid Dynamics (CFD) is an effective method for evaluating the impact of flow diverters (FDs) on aneurysmal hemodynamics. Consistent with prior studies^8–12^, hemodynamic alterations are recognized as critical determinants of clinical outcomes following FD therapy. Based on the mechanisms of parent artery reconstruction and flow remodeling by FDs, researchers have proposed various hemodynamic parameters to quantify the efficacy of FD treatment for aneurysms: from reductions in aneurysmal inflow intensity (such as aneurysm inflow rate, average intra-aneurysmal flow velocity, and mean aneurysmal flow amplitude)^13–16^, to decreases in vascular pressure or stress (such as wall shear stress and pressure gradients)^8,17,18^, and to changes in intra-aneurysmal flow behavior (such as relative residence time and vortex core-line length)^11,12,19^. Among these, the reduction in intra-aneurysmal flow velocity has been recognized as an important hemodynamic parameter indicative of successful FD deployment.

Multiple clinical studies have confirmed the efficacy of adjunctive coil embolization with flow diverters^5,20,21^. Compared to standalone FD deployment, PED combined with coil embolization achieves a higher occlusion rates, with mid- to short-term complete occlusion rates ranging from 72% to 94.4%, while significantly reducing retreatment rates. However, appropriate packing density in the flow diverters assisted coiling cases has been always concerned clinical issue. Zhang et al.^*5*^ suggested that FD-assisted coiling can improve the postoperative hemodynamic environment with lossen packing. Loose packing is sufficient as long as the hemodynamics within the aneurysm stabilize. They found that when the coil packing density reached an average of 7.06% or after the placement of a second coil, the hemodynamics within the aneurysm stabilized, making further coiling unnecessary. However, this large-aneurysm finding may not extend to small-to-medium aneurysms. Damiano et al.^22^ used finite element modeling to identify 11% PD as the hemodynamic saturation threshold beyond which additional coils yield diminishing returns. The results in this study demonstrate that the deployment of the TB flow diverter alone substantially alters the hemodynamic environment within the aneurysm sac, including reductions in inflow volume, average flow velocity, wall shear stress (WSS), and residual flow volume (RFV). These changes are further enhanced by adjunctive coil embolization. In addition, the study reveals that once the coil packing density reaches 5% for small and medium-sized aneurysms, the improvements in hemodynamic parameters (e.g., inflow volume, average aneurysm flow velocity, WSS, normalized WSS, and RFV) plateau, with no statistically significant benefit observed beyond this threshold.

Insufficient reduction in blood flow velocity at intra-aneurysmal and neck, as well as the persistence of jet flow, are prognostic factors for incomplete healing after cerebral aneurysm treatment. FD combined with loose coil packing can promote complete aneurysm healing while reducing complication rates and the need for retreatment. This could assist physicians performing TFD implantation for intracranial aneurysms (IAs) in determining the optimal number of coils needed to achieve the best treatment outcomes from a hemodynamic perspective, ultimately supporting optimized clinical decision-making.

This finding challenges the high-density packing employed in simple coiling or conventional stent-assisted coiling. High density packing is associated with increased procedural risks, including intraoperative rupture^23,24^ and higher costs^25^. A loosing packing strategy (approximately 5% PD) is sufficient when combined with TB deployment, achieving effective hemodynamic stabilization without the need for excessive coil insertion which consistent with our clinical outcome. However, whether the specific 5% packing density is valid or not which should be verified in the future study.

From a clinical perspective, this study underscores the importance of striking a balance between achieving hemodynamic stabilization and minimizing procedural complications. Excessive intrasaccular manipulation associated with high dense coil packing increases the risk of aneurysm rupture, particularly in small and medium-sized aneurysms with fragile walls. Additionally, the financial burden of dense coiling can be significant, especially in resource-limited settings. By identifying 5% PD as the threshold for optimal hemodynamic improvement, this study preliminarily provides a practical guideline for clinicians to maximize efficacy while minimizing risks and costs.

## Limitations and future directions

While the study offers robust evidence through patient-specific modeling and advanced simulation techniques, certain limitations should be acknowledged. First, the sample size, though reasonable (26 patients with 29 aneurysms), may limit the generalizability of the findings to larger or more diverse populations. Second, the study focuses on anterior circulation small and medium-sized aneurysms (diameter 5–15 mm) therefore, some confounder factors can be controlled. Due to the number of cases included was limited, and there was no further grouping, such as aneurysm location, morphology, or neck size. The applicability of the conclusions of this study will be observed in different types of aneurysms in the future. Third, computational models, while highly accurate, cannot fully replicate the complex biological processes involved in aneurysm healing, such as endothelialization and thrombosis. Fourth, the non-Newtonian rheology, patient-specific inflow conditions, and fluid-structure interaction (FSI) providing a more physiologically precise representation of the hemodynamic environment were not be incorporated. In this study, a comparative analysis of the relative changes in hemodynamics induced by varying coil packing densities under identical modeling assumptions were investigated. A simplified Newtonian fluid model and rigid walls as a well-established approximation commonly used in numerous prior CFD studies of intracranial aneurysms^5,26^. This approach allows us to efficiently isolate and compare the effects of the intervention (coiling density) across multiple scenarios while controlling for other variables. This simplification may affect the absolute values of hemodynamic parameters like WSS; However, the trends and comparative differences between the groups, which are the focus of the conclusions, remain valid and informative. About patient specific inflow conditions and rigid vessel walls, these are issues worth analyzing^27^. In future studies, the impact of these factors will considered on hemodynamics after aneurysm embolization, and further improve the research conclusion. In the our previous study focus on hemodynamic analysis of vertebral artery aneurysm treated flow diverter assisted coiling, grid verification has been carried out, and considering the convergence and the consumption of computing resources, the corresponding grid sizes for different devices are proposed^28^. Fifth, in this study, the reduction ratio of parameters was used as a standardized parameter for comparison. Thus, deviations from the inflow boundary conditions should be minimized. Finally, the coil configuration effect was not considered.

Future studies could expand on these findings by incorporating larger cohorts and examining long-term clinical outcomes, including aneurysm occlusion rates and recurrence. Additionally, exploring the effects of coil packing density in conjunction with other flow diverters or in different aneurysm morphologies could provide further insights into optimizing treatment strategies.

## Methods

### Patient selection

Between September 2020 and December 2022, 29 anterior circulation saccular small and medium-sized aneurysms (diameter 5–15 mm) from 26 patients treated with a TB flow diversion device or combination with adjunctive coil embolizaition were consecutively enrolled in Huashan Hospital Affiliated to Fudan University and Jing’an District Central Hospital. The study was performed in accordance with the Declaration of Helsinki. The Institution Review Board of Huashan hospital affiliated to Fudan university approved this retrospective study and waived the requirement for informed consent. Finite element analysis was employed to simulate the hemodynamics preoperatively, post-implantation of the TB device alone, and at adjunctive coil embolization rates of 5%, 7%,10%, 15%, and 25% following the TB deployment. This analysis included five scenarios to assess variations in key hemodynamic parameters including inflow rate (Q_inflow_), averaged aneurysmal flow velocity (Va), wall shear stress(WSS), normalized wall shear stress (NWSS), and normalized and ratio residual blood volume (nRFV and rRFV) under different packing density thresholds (0.1 m/s, 0.15 m/s). The goal was to identify the optimal packing density through variance analysis and multiple comparisons.

Relative change, quantifying the effectiveness of different packing densities in reducing hemodynamic parameters, was defined as follows:

A = (Pre-operation - TB device alone)/Pre-operation.

B = (Pre-operation − 5% embolization)/Pre-operation.

C = (Pre-operation − 7.5% embolization)/Pre-operation.

D = (Pre-operation − 10% embolization)/Pre-operation.

E = (Pre-operation − 15% embolization)/Pre-operation.

F = (Pre-operation − 25% embolization)/Pre-operation.

### Model reconstruction and FEM simulation

The patient-specific aneurysm models were reconstructed based on 3D-DSA images and exported in STL format. The operations of trimming and smoothing for aneurysm model were carried out in Geomagic Wrap 2015 (Research Triangle Park, North Carolina, USA) to remove unnecessary minor branches.

Adopting the FEM approach established in a previous study^29^, we performed the virtual implantation of the stent and coil. The first step was to generate the flow diverter (Tubridge) and coil models required for subsequent simulations, which was performed by NX 12.0 (Siemens PLM Software, Plano, TX, United States) and MATLAB (MathWorks, Natwick, MA). The simulation of Tubridge was divided into three stages: compression, delivery, and deployment, all of which were realized by using ABAQUS version 6.14 (SIMULIA, Providence, Rhode Island, USA). The landing zone of Tubridge was determined by the postoperative angiographic images. And the “pull-and-push” simulation of coil was also conducted in ABAQUS, which was pulling the coil into the microcatheter, and pushing it into the aneurysm sac in detail.

### CFD simulation and hemodynamic analysis

**Fig. 5:**
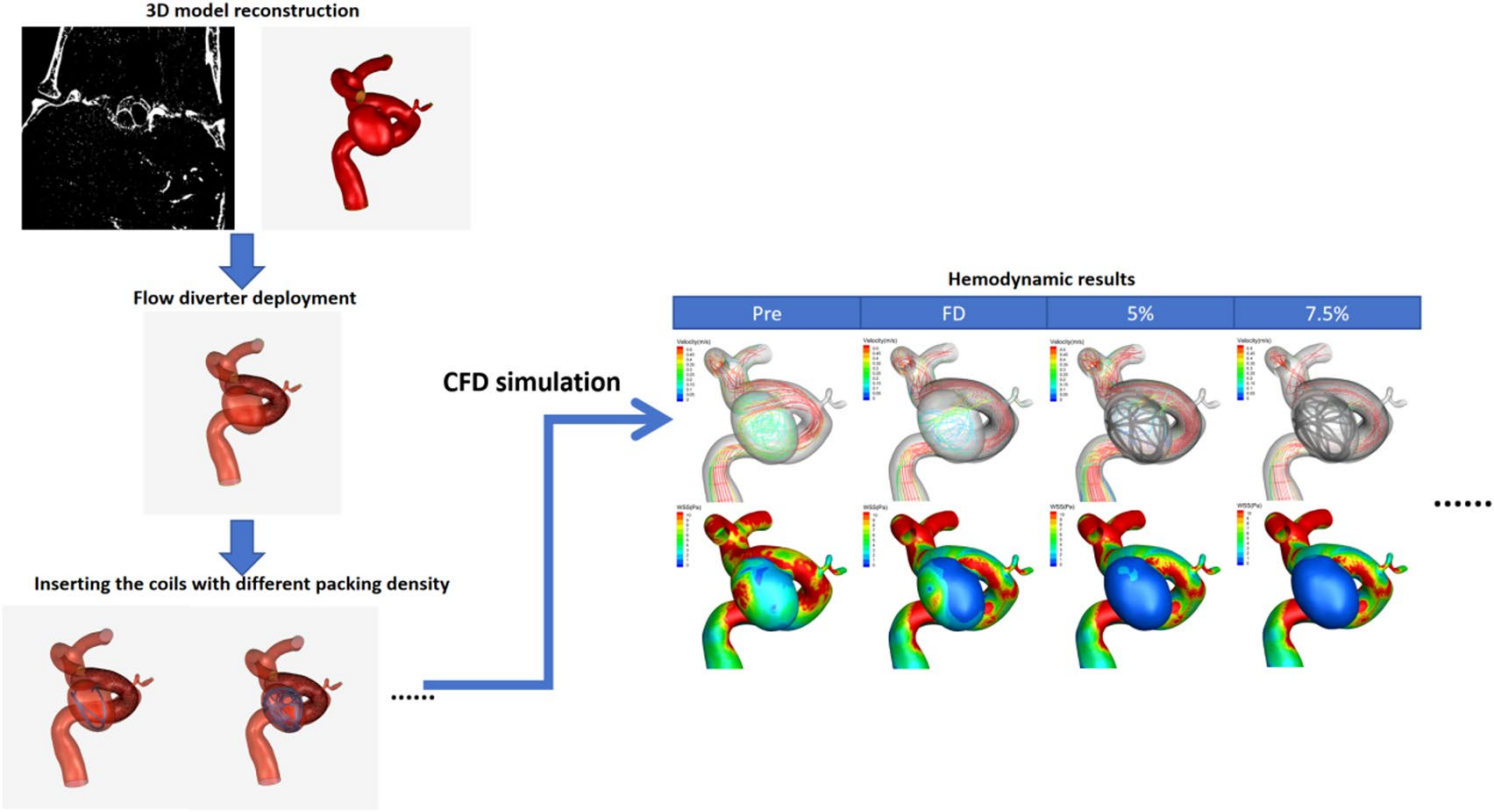
Flow chart. The reconstructed aneurysm model and the finite element models of the Tubridge and coils were used for hemodynamic simulation.

The reconstructed aneurysm model and the finite element models of the Tubridge and coils were used for hemodynamic simulation (Fig. 5). In this study, multiple CFD simulations were performed for each aneurysm, including preoperative baseline model and Tubridge-coil models with coil packing density (PD, the ratio of the coil volume to the aneurysm sac volume) from 0 to 25%, incremented at 2.5% intervals in PD. The 2.5% increment in PD was selected based on Zhang’s research^5^, which showed an increase in PD of about 3% for each additional coil, so a more conservative value of 2.5% was used in this study. Firstly, the aneurysm, Tubridge and coil models were imported into ANSYS ICEM CFD version 16.2 (ANSYS Inc, Canonsburg, PA, USA) for meshing, with a global mesh size of 0.16 mm. The surface mesh size of Tubridge was set to 1/6 of the wire circumference, which was 0.018 mm, and the mesh size of the coils was set to 0.1 mm^28^. Then, the ANSYS CFX version 2019 (ANSYS Inc, Canonsburg, PA, USA) was employed to perform the CFD simulation based on the Navier-Stokes equations. Several assumptions were adopted in this study. Blood was modeled as an incompressible, laminar, Newtonian fluid with a density of 1056 kg/m³ and a viscosity of 0.0035 kg/m·s. The vessel wall was assumed to be rigid and no-slip. All computational cases only utilized one ICA as the inlet. Outlets included the M1 segment of the MCA, the A1 segment of the ACA, and the ophthalmic artery. If the images clearly showed the posterior communicating artery and the anterior choroidal artery, these two locations were also considered as outlets. The inflow rate was set to 4.6 ml/s, and the all outlet flow rates were calculated according to the Murray flow rate distribution law^30^.

In this study, hemodynamic parameters including the inflow rate at the neck (Q_inflow_), the average velocity of the aneurysm sac (Va), the WSS of the aneurysm, the normalized WSS (NWSS), and the normalized residual flow volume (nRFV) were calculated with average flow velocities of greater than 0.1 m/s, 0.15 m/s, respectively. The rRFV was defined as the ratio of the RFV to the aneurysm sac volume. Subsequently, the reduction ratio of the Q_inflow_, Va, and nRFV was calculated to describe the flow changes after Tubridge deployment and coils embolization.

### Statistical analysis

The statistical analysis was performed using SPSS 25.0 (IBM Corp, Chicago, IL, United States). Normal distribution was confirmed for each continuous variable before the tests. One-way ANOVA was used to evaluate the reduction ratio of the calculated parameter for each PD embolization. We also used the regression models to filter the variables that were not collinear and then fit the relationship between PD and those variables. A p-value < 0.05 was considered statistically significant.

## Conclusion

This study demonstrates the hemodynamic benefits of adjunctive coil embolization in the treatment of small and medium-sized anterior circulation aneurysms with the Tubridge flow diverter. The preliminary findings indicate that a coil packing density of 5% might be sufficient to achieve significant hemodynamic improvements, with no additional benefit from denser packing.

## Data Availability

The raw data supporting the conclusions of this article will be made available by the authors, without undue reservation.
